# Snowfall in the Northwest Iberian Peninsula: Synoptic Circulation Patterns and Their Influence on Snow Day Trends

**DOI:** 10.1155/2014/480275

**Published:** 2014-07-24

**Authors:** Andrés Merino, Sergio Fernández, Lucía Hermida, Laura López, José Luis Sánchez, Eduardo García-Ortega, Estíbaliz Gascón

**Affiliations:** University of León, Instituto de Medio Ambiente (IMA), 24006 León, Spain

## Abstract

In recent decades, a decrease in snowfall attributed to the effects of global warming (among other causes) has become evident. However, it is reasonable to investigate meteorological causes for such decrease, by analyzing changes in synoptic scale patterns. On the Iberian Peninsula, the Castilla y León region in the northwest consists of a central plateau surrounded by mountain ranges. This creates snowfalls that are considered both an important water resource and a transportation risk. In this work, we develop a classification of synoptic situations that produced important snowfalls at observation stations in the major cities of Castilla y León from 1960 to 2011. We used principal component analysis (PCA) and cluster techniques to define four synoptic patterns conducive to snowfall in the region. Once we confirmed homogeneity of the series and serial correlation of the snowfallday records at the stations from 1960 to 2011, we carried out a Mann-Kendall test. The results show a negative trend at most stations, so there are a decreased number of snowfall days. Finally, variations in these meteorological variables were related to changes in the frequencies of snow events belonging to each synoptic pattern favorable for snowfall production at the observatory locations.

## 1. Introduction

Snow and ice, together with windstorms, are among the most important meteorological hazards in winter. In fact, heavy snowfall can significantly inconvenience users of the transportation infrastructure and can increase the number of injuries and fatalities related to traffic accidents [1]. In mountainous areas, avalanches caused by snow accumulation are a potential danger, whose trigger mechanisms are difficult to identify [2]. Another source of difficulty is wet snow icing accretion on power lines, causing failures of high and medium voltage power supplies [3]. Moreover, heavy snowfall followed by melting can trigger large floods that represent a serious natural hazard [4]. Furthermore, the accumulation of snow in mountain headwater basins is a major water source, particularly in semiarid environments [5]. Additionally, snowfall permits the development of economic activities related to winter tourism in mountainous areas [6].

Snowfall detection and measurement represent very difficult problems in modern hydrometeorology. For snowfall detection, it is necessary not only to consider precipitation systems, but also to determine other variables such as the level of snowflake fusion (melting level), in order to discriminate precipitation reaching the ground as rain or snow. Determining the melting level is also critical for hydrologic models, since it determines the basin surface exposed to rain and the possibility of flooding. Ground measurements are complicated owing to detection technology limitations, snow drift and accumulation issues, and error definition [7].

Usually, the melting level is slightly below the freezing level, which is the height of the 0°C isotherm. Lundquist et al. [8] found that, in California at 1.5°C, 50% of the precipitation falls as rain and 50% as snow. Similar results were found in the Alps [9], the Bolivian Andes [10], and Sweden [11]. Nevertheless, at times complex topography favors low-level thermal inversions that cause superposition of warm layers atop cold ones, which complicates the identification of precipitation type.

In recent decades, several studies have shown the influences of climate change on water resources availability, precipitation, and temperature [12, 13]. In the Iberian Peninsula, it has been shown that temperature during winter months has increased in several mountainous regions [14]. Furthermore, since temperature is expected to continue rising globally, it is reasonable to expect a decrease in snowfall frequency and accumulated snow depths [15, 16] and, therefore, shorter duration of the snow season [17, 18]. However, there is little information about how future heavy snowfall events might evolve. Changnon [19] reported less frequent but more intense snowstorms in the USA. López-Moreno et al. [20] studied the frequency and intensity of snowfall events in the Pyrenees (Spain), finding a decrease in snowfall intensity at elevations below 1000 m above sea level (asl) and increased intensity above 2000 m asl. Pons et al. [21] analyzed snowfall frequency in the northern Iberian Peninsula, finding a 50% decrease in the number of snowfall days since the mid-1970s. These authors stated that the decrease in snowfall days was associated with increasing temperatures, especially at low-elevation stations. However, for stations at high elevations, the main factor related to the decline in number of snowfall days was a decrease in overall recorded precipitation [22].

Synoptic scale patterns responsible for severe snowfall episodes have been used in many mountainous areas to define atmospheric conditions under which these phenomena occur [23, 24]. Esteban et al. [25] characterized synoptic situations producing heavy snow in the eastern Pyrenees, concluding that northwest flows with strong isobaric gradient were most conducive to heavy snowfall.

In this paper, we analyze synoptic scale conditions favorable for the most important snowfall in the principal cities of the northwestern Iberian Peninsula. Once we establish the synoptic scale patterns, we study the frequency of each and their temporal evolution, as well as the trend in number of days with recorded snowfall. Finally, since the number of snowfall days has decreased, we assess whether this negative trend is attributable to changes in large-scale circulation patterns, to variations of temperature, or to a combination of the two.

## 2. Study Area and Databases

**Figure 1:**
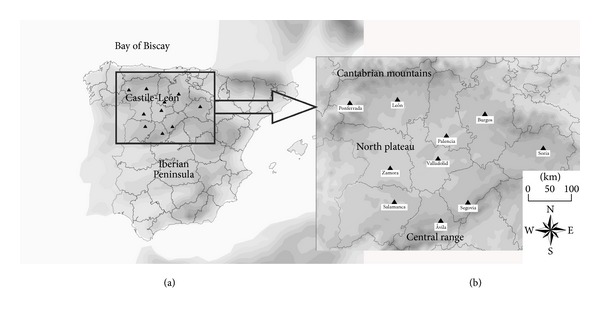
(a) Iberian Peninsula. (b) Study area of Castilla y León and locations of its principal cities.

**Figure 2:**
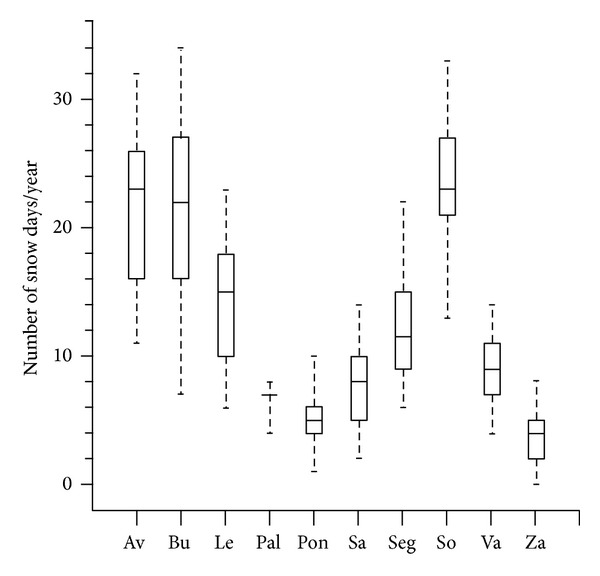
Box and whisker plot for number of snow days/year for each station (Ávila, Burgos, León, Palencia, Ponferrada, Salamanca, Segovia, Soria, Valladolid, and Zamora). The lower end of the box indicates the 25th percentile, the central line indicates the median, and the line above the box indicates the 75th percentile. The error lines above and below the box indicate the 95th and 5th percentiles, respectively.

The northern plateau region is in the northwest Iberian Peninsula (Figure 1). This region is characterized by a central plateau with elevation about 800 m asl, surrounded by mountain ranges around 2000 m asl, except for the southwest and northeast where elevations are lower. The Iberian Peninsula is often affected by the polar jet stream, resulting in a variety of situations under which precipitation occurs in the study area. However, snowfall is more frequent in mountainous areas because of upslope moisture flow producing a precipitation blocking effect. Thus, snowfall frequency is therefore significantly reduced toward the center of the northern plateau. Thereby, cities with more snowfall days are near mountain ranges, owing to their high altitude and influence of the mountains (Figure 2).

To classify situations conducive to snow in Castilla and León, we selected the most significant snow events in 10 major cities of the region between 1960 and 2011. The choice of these observation sites was made because most of the regional population is concentrated near these cities, which are also in areas more vulnerable to snowfall. Moreover, long-term, reliable time series are available from stations at these sites. We also selected stations overseen by a professional staff and, where possible, airport weather stations, because these are usually at very stable locations. The criteria for snowfall events selection were days with more than 5 mm of accumulated precipitation and temperature below 2°C. According to Lundquist et al. [8], the accumulation of snow is greater than snowmelt at temperatures less than 2.5°C. In addition, stations with the presence of observers always report snow days under these conditions. A total of 241 snowfall events met these criteria for at least one of the 10 stations.

## 3. Methods of Investigation

### 3.1. Establishment of Synoptic Patterns

Synoptic conditions of snow events were characterized by extracting temperature and geopotential height at 850 and 500 hPa from NCEP-NCAR reanalysis data [26] of the 241 events in the database, over the region 30–60°N by 30°W–10°E with 2.5° resolution. These variables describe synoptic scale atmospheric conditions under which the snowfall events developed. The 850 hPa and 500 hPa geopotential heights show atmospheric circulations in the lower and mid-troposphere. These fields have been evaluated by several authors to define synoptic patterns of heavy snow events [25, 27]. The 850 hPa and 500 hPa temperatures provide a good approximation of the snow level and therefore permit determination of areas (according to their elevations) that may be affected by snow. Other atmospheric fields (sea level pressure and 1000–500 hPa thickness) used in previous studies [25] to define synoptic patterns of heavy snow were evaluated by the scree test [28]. It was found that addition of sea surface pressure and 1000–500 mb thickness fields did not provide more meaningful factors. Window dimensions were selected to be consistent with the synoptic scale analysis of the present study and to avoid circulation features in regions remote from the study area. Time was standardized for all snow events. Because synoptic conditions showed little change during the event, NCEP data files at 12:00 UTC were chosen as the most representative.

Synoptic scale patterns were produced from reanalysis data using principal component analysis (PCA) and cluster techniques (CT) based on retained principal component loadings. Days possessing similar loadings on the extracted components were clustered together. Both methods have been widely used for classifying and establishing weather patterns [29, 30].

PCA is designed for the reduction of the number of variables while maintaining strong representation of variability contained in the original data. PCA is a method that ensures that only the fundamental variation modes of the data are considered in the clustering process [31]. In the present study, PCA was applied with a correlation matrix in* T*-mode where the variables are the selected days and grid points as observations. The number of components explains a significant proportion of the total variance [32]. Here, we chose only the most important extracted components that account for at least 90% of the total variance. Additionally, the number of components extracted for each variable was evaluated via the scree test [28], and the rule of thumb of North et al. [33] was used for estimating the sampling error. PCA provides component scores, which are standardized values representing the relationship between observations and retained orthogonal components.

The CT allows for separation of data into groups, whose identities are previously unknown. In this study, the selected CT was the nonhierarchical* K*-means method [34]. The Euclidean distance was chosen for classifying groups of data according to their similarity. One of the sources of subjectivity in this method is the requirement that the number of final conglomerations (*k*) is predetermined ahead of time. The selection of* k* can be done objectively by computing the minimum decrease of intragroup distances. Nevertheless, the decision regarding the number of groups is not a completely objective task since a degree of subjectivity is present, based on researchers' experience [35]. Previous works have described the application of these statistical techniques toward determining weather patterns in detail [36].

### 3.2. Trend Analysis

Once the synoptic patterns favorable for the most significant snow events have been identified, we test how frequency variations of snow events in the observed patterns affect the number of snowfall days recorded at each station. To this end, we analyzed trends in the number of snow days (days on which solid precipitation occurred during the study period) between 1960 and 2011 at the stations used previously to extract the synoptic patterns. We further investigated trends in precipitation and temperature at the selected stations. Our aim was to determine whether the decrease in frequencies of snow events belonging to each aforementioned pattern was due to climatic variability patterns or to an increase of temperature favoring increased melting level altitude. As already noted, despite the presence of patterns favorable for precipitation, there might be no snowfall at the stations.

We should first point out that measurements at weather stations can be easily affected by nonclimatic factors such as changes in instrumentation, exposure or measurement technique, observers, observation times, or the use of different methods to calculate monthly averages [37]. Environmental changes at the station location should also be taken into account, with emphasis on growth of urbanized areas [38]. According to Conrad and Pollak [39], a numerical series representing variations in climatological elements can only be defined as homogeneous if such variations are caused exclusively by time or climate changes. Thus, prior to trend analysis, we assessed homogeneity of the time series data for all stations, using the nonparametric Kruskal-Wallis test [40, 41].

Subsequently, statistical techniques of the nonparametric Mann-Kendall test were applied at the 10 stations, to determine trends in number of snowfall days and their statistical significance. These techniques were applied using the MAKESENS template [42]. Sign of the trends was tested using the Mann-Kendall test, whereas slope of the linear trend was estimated using Sen's method [43]. The main limitation of the Mann-Kendall trend test occurs when there is serial correlation of the data in space and time. Climatological data do not normally satisfy the condition of independent and identically distributed samples, so the existence of positive serial correlation increases the probability that the test detects a trend when there is none (false positive). This would lead to rejection of the null hypothesis when it is actually true. A negative serial correlation, however, decreases the possibility of null hypothesis rejection [44].

To assess this effect, we executed the method proposed by von Storch and Navarra [38], to replace the original time series. If there is no correlation between successive observations, the null hypothesis is accepted, the data are independent, and there is no persistence in the time series. Otherwise, before applying the Mann-Kendall test, we performed prewhitening via the method of Tabari and Talaee [45].

The Mann-Kendall test is used in climate studies to detect trends in data series [45, 46], by statistically determining whether the values of a variable are increasing or decreasing over a period of time [47]. This test is a nonparametric statistical method; that is, no distribution model is assumed for the random variables studied. Other advantages of this test include its low sensitivity to sudden breaks owing to inhomogeneity of the time series [48] and the fact that time series containing missing values may be used. The test consists of comparing the value of a given variable with its value at a prior time and assigning a positive or negative sign depending on whether the later value is smaller or larger than the earlier one. A zero statistic indicates no change in the trend with time, so the null hypothesis is accepted. The greater the deviation from zero is, the greater the data trend is [47]. Positive values indicate an upward trend and negative ones a downward trend.

Finally, to estimate the magnitude of change per unit time of a linear trend, we used the nonparametric Sen's slope estimator [48]. This method obtains the slope for each possible pair of measurements in different years; the median of all slope values obtained is the estimator.

## 4. Results

### 4.1. Synoptic Patterns Favorable for Snowfall in Castilla y León

As noted above, applying PCA for geopotential height and temperature at 500 and 850 hPa, respectively, we extracted 5, 5, 8, and 7 components. These components were extracted until reaching 90% of explained variance for each variable. These extracted components were evaluated by means of the scree test [28], and the rule of thumb of North et al. [33] was used for estimating the sampling error (Figure 4).

Then, with a matrix of a total 25 PCA results (columns) and 241 study days (rows),* K*-means clustering was applied. Intragroup distances were computed for *k* = 2, 3, 4,…, 20 and showed a minimum decrease for *k* = 4, considered the optimal cluster number. For this result, we classified a total of 84, 71, 30, and 56 events in each cluster. To physically interpret the results, we calculated mean atmospheric fields for each cluster, averaging data grids corresponding to the events grouped in each cluster (Figures 5–8).

**Figure 3:**
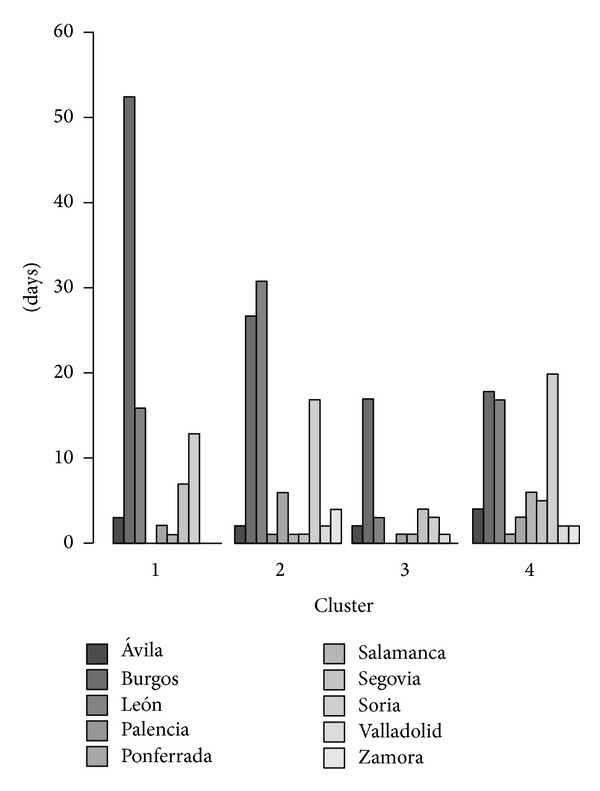
Cluster distribution of snow events by city between 1960 and 2011.

**Figure 4:**
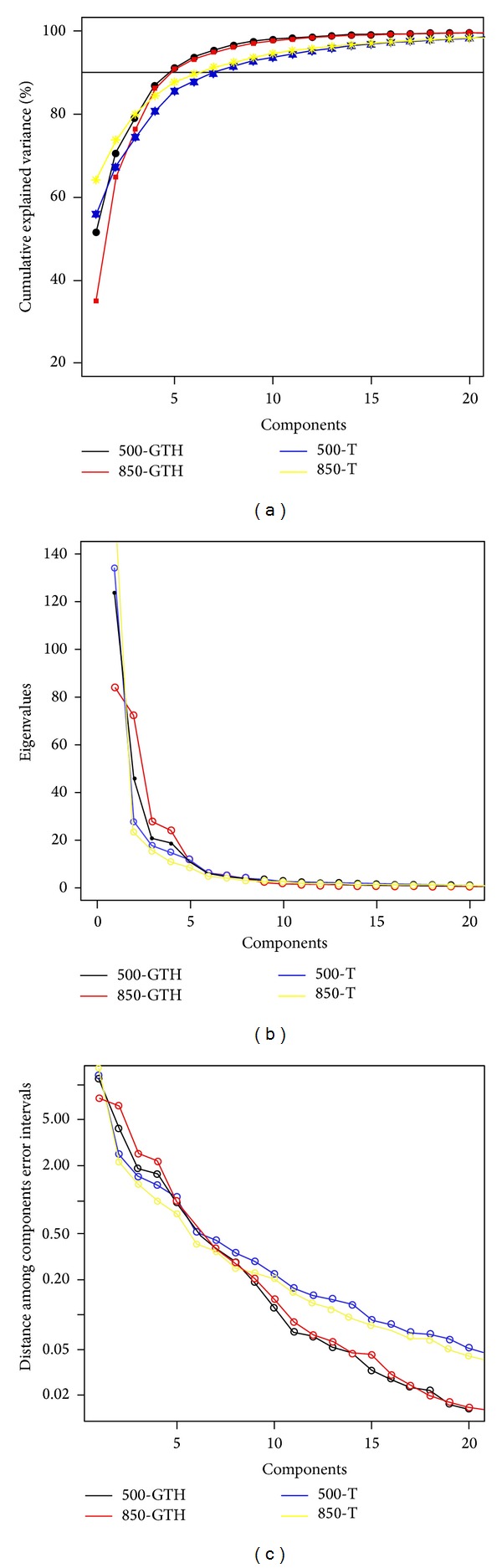
PCA applied to each variable. (a) Cumulative explained variance (%); black horizontal line indicates cumulative variance 90%. (b) Scree test. (c) North rule of thumb.

Figure 3 represents the cluster distribution at each station analyzed. The results show that the cities of Palencia, Valladolid, and Zamora, all at low altitude and far from mountainous areas, have a lower frequency of snow events. We call the 4 clusters favorable to snowfall in the region “Arctic advection,” “western flow,” “northeastern advection,” and “cyclonic circulation.”

#### 4.1.1. Cluster 1: Arctic Advection

**Figure 5:**
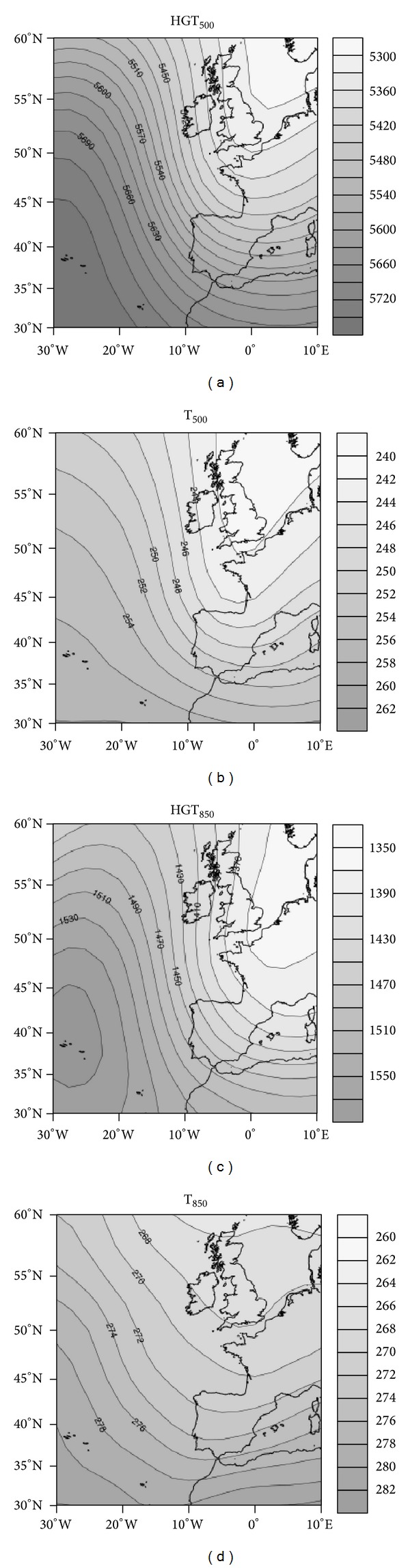
Synoptic environment of cluster 1. Geopotential height (HGT) and temperature (T) at 500 hPa (a, b) and 850 hPa (c, d).

Cluster 1 is defined by the arrival of maritime Arctic air over the Iberian Peninsula. An upper-air ridge associated with a surface high in the Atlantic, coupled with a trough in western Europe, produces a strong barometric gradient that causes horizontal advection of maritime Arctic air over the peninsula. With this configuration, a cold tongue at mid-levels settles over the northwest peninsula, whereas at low levels, the maritime flow generates significant cold and moist air advection over the northern peninsula (Figure 5). This configuration usually produces orographic precipitation at the windward base of mountain ranges, in this case mainly affecting the Cantabrian region. In the study area, the Cantabrian Mountains in the north orographically block moisture, resulting in precipitation in nearby areas. However, to the east the Cantabrian Mountains have lower elevations, favoring the entry of clouds and moisture to this region. Moreover, the cold air mass at upper levels causes greater instability, which increases the possibility of convective precipitation. Thus, the city of Burgos had more events of this nature, owing to the entry of clouds from the eastern part of the Cantabrian Mountains. This affects Segovia and Soria to a lesser extent, with 7 and 13 events, respectively (Figure 3). Despite being on the southern slopes of the mountain range, the city of Leon had 16 such events. This is explained by the proximity of the city to the mountains and suggests strong moisture advection events that transport clouds toward the southern face of the mountain range.

#### 4.1.2. Cluster 2: Westerly Flow

**Figure 6:**
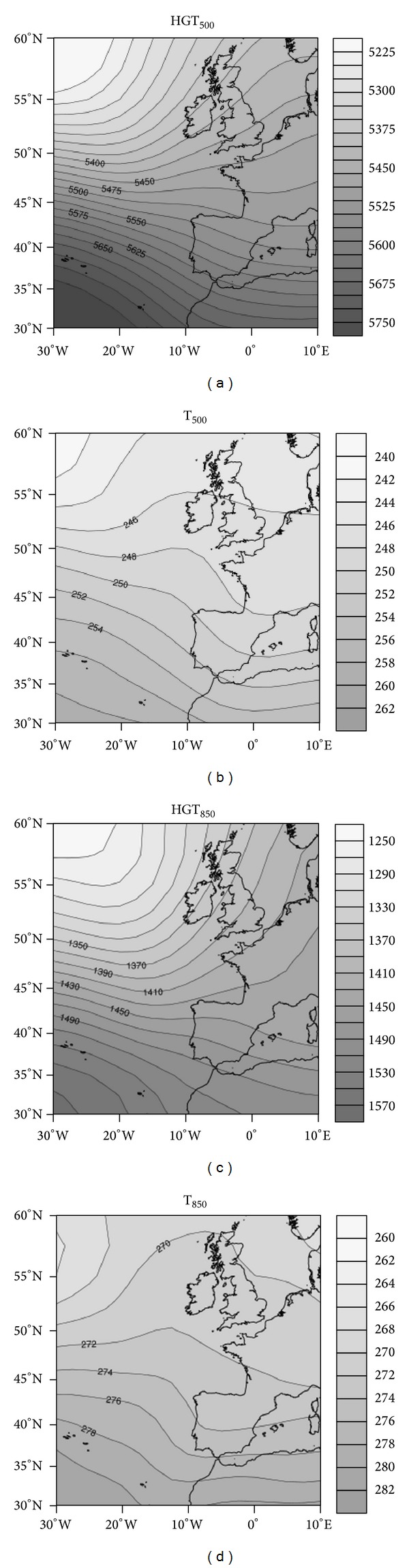
As in Figure 2 but for cluster 2.

Cluster 2 is marked by the movement of storms across the North Atlantic, while the tropical ridge shifts to the southwest peninsula. Thus, an intense west-northwest barometric gradient that advects the Northwester Atlantic air is established over the peninsula. This air mass has a long maritime trajectory, acquiring high humidity and gradually warming at lower levels, owing to contact with the ocean. This is seen from 850 hPa temperatures (Figure 6). This situation is favorable for the entry of cold fronts from the northwest of the Iberian Peninsula that produce abundant precipitation in the study area. However, cold fronts are often accompanied by increased melting level because of warm air advection ahead of the fronts, since cold advection occurs after frontal passage. Therefore, for this situation, to produce snow at relatively low levels, there must be earlier cold advection over the Atlantic, which is subsequently transported ahead of the front.

The northwest regions are in Castilla y León, and those at higher altitude are most affected by snowfall events since the melting level is relatively high. The cities of Ponferrada and León had conditions resulting in most snowfall events (Figure 3). Burgos and Soria also had numerous such events under these conditions, owing to their high altitudes (about 1000 m).

#### 4.1.3. Cluster 3: Northeasterly Advection

**Figure 7:**
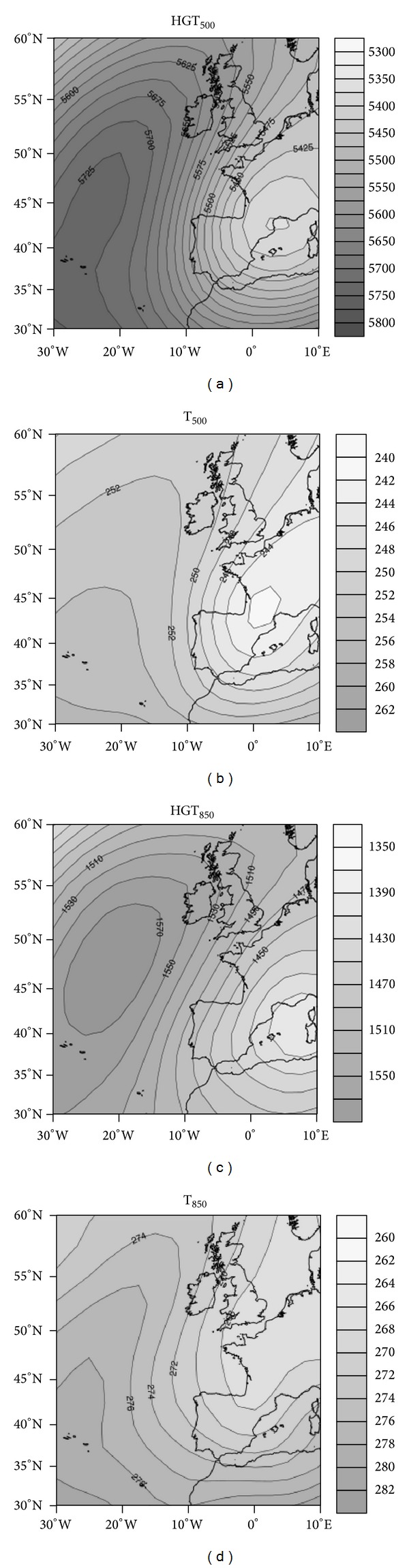
As in Figure 2 but for cluster 3.

The conditions described by cluster 3 correspond to arrival of a continental polar air mass over the Iberian Peninsula. A low-pressure system is situated over the western Mediterranean and an upper-air ridge over northwestern Europe. This forms a strong northwest barometric gradient that advects continental polar air. Very cold isotherms are evident under these conditions, at both 850 hPa and 500 hPa (Figure 7). This is because the flow originates from East Europe, where the continental air mass produces the very cold temperatures and low moisture content characteristic of winter. Because this air mass is very dry, precipitation in the study area should not be abundant. However, the brief transit of the air mass over the Bay of Biscay supplies moisture conducive to precipitation upon reaching the Cantabrian Mountains. The northeast flow generates lingering clouds over the northern face of the Cantabrian range. This causes precipitation in this area and further east, within a corridor formed by the Cantabrian Mountain. Although precipitation is often weak, persistent snowfall together with low temperatures can result in significant accumulation. Burgos clearly stands out in this regard, with 17 snowfall events. Other cities to the east lagged far behind, such as Segovia and Soria where there is a general deficiency of moisture, thereby preventing significant snowfall (Figure 3).

#### 4.1.4. Cluster 4: Cyclonic Circulation

**Figure 8:**
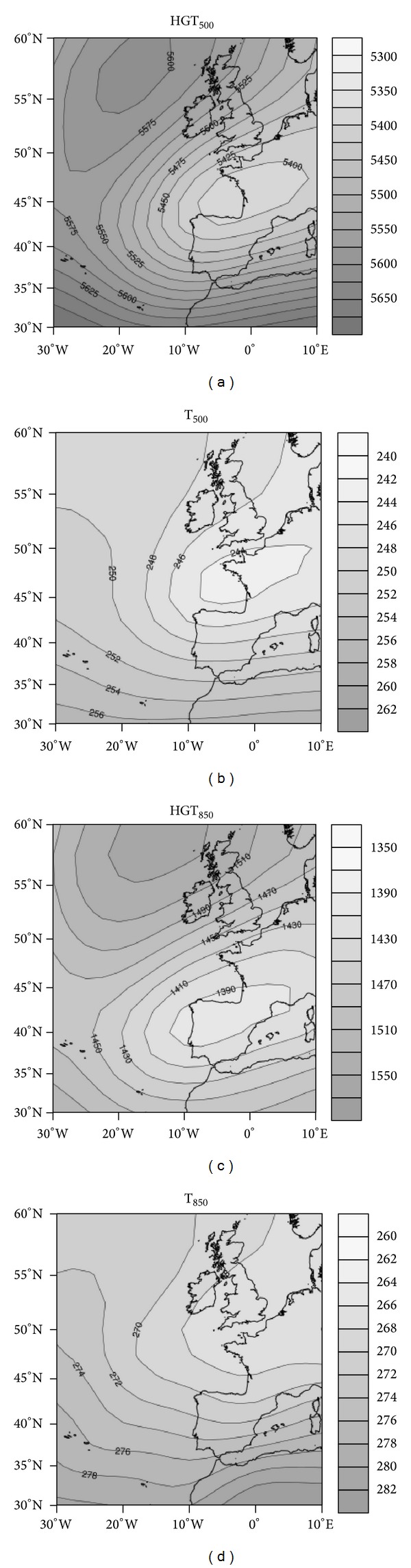
As in Figure 2 but for cluster 4.

Cluster 4 shows a cyclonic circulation at relatively low latitudes, affecting the Iberian Peninsula. By contrast, there is an anticyclonic circulation area in the North Atlantic, where there are typically passing disturbances (Figure 8). This circulation produces a strong thermal gradient over the study area, since there is a collision of air masses of different characteristics over the peninsula. These include a very cold and dry continental polar air mass and a warm and moist maritime tropical air mass. With this situation, heavy snowfall occurs within a narrow swath where these air masses collide. The location of disturbances determines the region of this collision and resultant snowfall. This configuration produces most of the snowfall in the region, since such air mass collisions can occur over the center of the plateau. Notably, over 50% of snowfall events under these conditions occur in the city of Salamanca. However, the majority of such events are in the cities furthest north and at highest elevation, since these are nearer to the cold air mass (Figure 3).

### 4.2. Synoptic Pattern Trends and Climatic Variability

**Figure 9:**
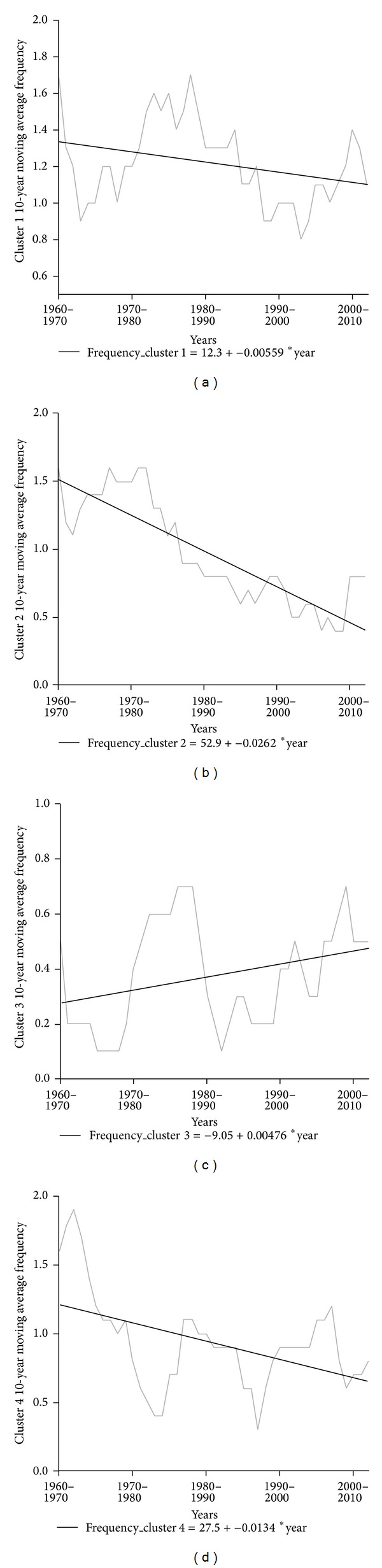
Ten-year moving average frequency of snow events. Cluster 1 (a), cluster 2 (b), cluster 3 (c), and cluster 4 (d).

To determine the temporal evolution of snow events frequencies observed in each cluster, we plotted 10-year moving average frequency for the snow events recorded in each cluster. The linear regression trends were computed with the original unsmoothed data to avoid the serial correlation induced by the moving average smoothing.  Figure 9 shows a clear decline in events with a western flow pattern, which in recent years have declined from an annual average of 1.5 to 0.5. For the configuration described by the cyclonic circulation pattern, there was also a clear downward trend. Nevertheless, in this case, a series of cycles around 15–20 years is discerned, during which this pattern is more frequent.

The configurations describing the northeastern advection and Artic advection patterns had a very slight trend, without significant correlation at the 0.05 level. Thus, we cannot conclude that there were statistically significant changes in frequencies of these events. Therefore, in view of these results, we expect a decline in conditions favorable to snow precipitation in most of the cities analyzed. In particular, cities that correspond to the western flow pattern should show the greatest downward trend in number of snowfall days.

To extend the study of trends in snow days in each cluster, the nonparametric Mann-Kendall test was then applied to determine sign of observed trend and their statistical significance. The result of the Mann-Kendall test regarding the sign of the trend was −0.72 for cluster 1, −2.6 for cluster 2, 0.62 for cluster 3, and −1.33 for cluster 4. The trend was significant for cluster 2 at the 0.01 level. These results are consistent with the linear regression trends. A clear, significant, negative trend is observed for the western flow pattern. Meanwhile, trends in the northeastern and Arctic advection patterns are very weak and not significant. In addition, we analyzed seasonality of the snow days series by cluster. The autocorrelation function or correlogram was applied to the annual series of snow days by pattern, indicating no annual seasonality in any of the series.

Finally, some studies point to the connection between climate variability and teleconnection patterns, such as the North Atlantic Oscillation (NAO). This index uses the difference between the standardized subtropical Atlantic and subpolar Atlantic pressure values. Martín Vide and Fernández [49] found strong negative correlation between NAO values and precipitation on the Iberian Peninsula. This correlation was mainly on the central and southwestern peninsula, where rainfall depends upon storms from the southwest. However, Sáenz et al. [50] analyzed winter temperatures at stations in the northern peninsula, finding no statistically significant correlation with the NAO. As mentioned above, snowfall is also influenced by air temperature. Thus, López-Moreno and Vicente-Serrano [51] observed a positive trend of the NAO index and a decrease in frequency of weather situations producing snow accumulation in the Pyrenees. It is therefore expected that when the NAO index has negative values, there will be more frequent snowfall in the study region, although this is contingent on low-level temperatures.

Table 1 shows the average daily NAO index for snowfall events classified by cluster and for episodes producing snow at the stations within each cluster. In the total average, all patterns had a negative NAO. However, events classified with the cyclonic circulation pattern had larger negative values, whereas the average approached zero in the remaining clusters. This is because this configuration is marked by western flow at low latitudes and a positive pressure anomaly at high latitudes. In the remaining patterns, a meridional flow with high pressure dominates, encompassing the Atlantic.

Thus, a negative NAO favors the presence of a cyclonic circulation that produces precipitation across the study area. However, low temperatures are also necessary for snowfall. Therefore, we conclude that a strongly negative NAO greatly favors precipitation in the study area but not necessarily in the form of snow.

### 4.3. Snowfall Day Trends in Castilla y León

In this section, we examine the results obtained by analyzing trends in the number of snowfall days, maximum and minimum temperature, and precipitation.

The nonparametric Kruskal-Wallis test results indicate that all stations were homogeneous for all variables, except for the stations in the cities of Ávila (for snow days and maximum and minimum temperature) and Segovia (for maximum temperature). Serial correlation evaluation showed that the Burgos series had serial correlation for snowfall days and maximum temperature, the León station for minimum temperature, Ponferrada for snowfall days, and Salamanca for maximum temperature. In these cases, we performed prewhitening of the series prior to applying the Mann-Kendall test [45]. We decided not to include the Palencia station for number of snow days, because of a large number of missing values.

Table 2 shows trend results for each variable and station. A clear positive trend is evident for maximum temperature, which was the most significant trend at most stations. By contrast, there were no clear trends for minimum temperature, except for the two cities at the center of the plateau (Valladolid and Palencia), which had positive trends. These results are consistent with those of del Río et al. [52]. They observed no significant trends of minimum temperature during winter, whereas trends were positive and significant for maximum temperature in the central and southern plateau. Causes of these increases may be various forcings of the climate system such as the greenhouse effect, solar radiation, and aerosols [53, 54]. Nevertheless, there are works pointing to other external forcings, such as sunshine duration and cloudiness [55]. Variations in these parameters can be explained by changes of weather patterns from variations in teleconnection patterns that have stronger influence in winter, such as the NAO [56]. Increased sunshine duration and decreased cloudiness positively affect the variation of maximum temperatures. This effect is reversed for minimum temperatures, since these forcings favor nocturnal inversions. The predominant geomorphic unit in the study area is the plateau, which is very favorable for inversions, and may explain the low trends for minimum temperature.

For precipitation, most of the stations had a downward trend except in Palencia, Segovia, and particularly Ávila, where the trend was significant at 0.01 level. Studies similar to ours by Gallego et al. [57] showed a slight negative trend of precipitation for winter over 1954–2003. They noted an increase of light precipitation and a decline in moderate or heavy precipitation. The slight decrease of precipitation may be due to an increase in the NAO index during the second half of the 20th century [58, 59]. Thus, a positive trend of this index is consistent with precipitation reduction in the western Mediterranean during winter [58]. In this sense, many authors highlight a winter increase of synoptic patterns conducive to a lack of precipitation on the Iberian Peninsula [60, 61].

Consequently, broadly considering the rising temperatures and declining precipitation, negative trends in the number of snow days are expected. Correspondingly, all stations except Segovia had negative trends of this number. The downward trend was stronger and more significant in the western cities of Castilla y León, especially León. This result is consistent with the first part of this study, which outlined synoptic patterns responsible for heavy snow in each city. Thus, heavy snowfalls in León were very dependent on the western flow synoptic pattern, which has significantly declined in frequency during the second half of the 20th century. In contrast, eastern cities of the region, like Burgos and Soria, had heavy snowfalls under a variety of synoptic patterns characterized by flows with a northerly component. These patterns have had slight trends in frequency, which coincide with more moderate declines in frequency of snowfall days. The city of Segovia stands out, where there was a positive trend in the number of snowfall days. This may be because, together with Ávila, it is situated on northern slopes where light snow typically occurs owing to lingering clouds. Thus, these snowfalls are recorded as snow days, but they are usually weak and therefore not represented in the heavy snowfall database.

These results suggest that a decrease in number of snow days at the analyzed stations can be attributable to rising temperatures and decreased frequency of patterns favorable for heavy snow. This is in line with the results of Pons et al. [21]. Thus, the decrease in frequency of patterns conducive to heavy snow at the stations is largely attributable to increased low-level temperatures and, thereby, an increase of melting level. This is especially true in the western flow pattern, in which the freezing level was near the elevation at most stations, and therefore snowfall frequency at study-area stations has drastically decreased. Nevertheless, it should also be considered that an increase of the NAO index in winter months during the second half of the 20th century may be another cause for the decreased frequency of patterns favorable for heavy snow in the region.

## 5. Conclusions

Knowledge of the types of synoptic scale patterns leading to a particular meteorological phenomenon can aid in understanding of the causes of climate variability and more precise explanation of its changes in trend. In this work, we classified synoptic scale patterns defined by temperature and geopotential fields at 500 and 850 hPa, respectively, for 241 days with recorded important snowfall in at least one of 10 main observation stations in Castilla y León, from 1948 to 2011. By applying PCA and* K*-means cluster techniques, we obtained four patterns: Arctic advection, western flow, northeastern advection, and cyclonic circulation.

We obtained trends in number of snowfall days, maximum and minimum temperature, and precipitation for the stations from 1960 to 2011, using the Mann-Kendall test. The results show a significant decrease in number of snow days at stations in the northwest part of the region. We also found an increase in maximum temperatures and a decrease in precipitation although to a lesser extent. These results can be explained by conditions favoring snowfalls that dominate in an area. Thus, stations in the northwestern study area are very dependent on the western flow pattern for snowfall. This pattern is characterized by temperatures at lower levels that are not extremely cold. Consequently in recent years, there has been a pronounced decline in the number of snow events associated with this pattern. One possible cause is an increase of melting level, which has produced precipitation in the form of rain rather than snow at the stations.

However, cities in the eastern part of the region had the least negative trend for number of snow days. This area benefits from almost all the above patterns, especially those that generate advection with a northerly component. Thus, the decrease in frequency of snowfall events under these patterns was much smaller, because the melting level is low enough that a temperature increase fails to raise it above the station elevations.

Another possible cause of the decrease in number of snow days is an observed increase in the NAO index during winter months. Conditions conducive to snowfall at stations in the region are characterized by a neutral NAO, except for the cyclonic circulation pattern that has a clear negative NAO. Thus, at the Salamanca station, where this pattern occurs in more than 50% of snowfall events, this could be one cause of the significant decline in number of snow days.

Finally, only the Segovia station had a significant positive trend. The reason may be that it is the only station on the northern slope. Therefore, it commonly has lingering clouds from northerly advection, which produce snowfall days with only light precipitation.

As we have shown, changes in trends of meteorological phenomena respond to variations in large-scale atmospheric circulation patterns. In the special case of the snowfalls, they are directly affected by increased temperatures, and it is difficult to establish how much the reduced snowfall may be attributed to rising temperatures and how much to changes in weather patterns. In-depth study of the behavior of these patterns provides a basis for future analysis of trend changes in meteorological variables.

## Figures and Tables

**Table 1:** Average NAO index on heavy snow events, by cluster and station.

Cluster	1	2	3	4
Ávila	−0.30	−1.58	0.44	−0.79
Burgos	0.04	−0.12	0.06	−0.58
León	−0.22	−0.08	−0.50	−1.23
Palencia	N.A.	−0.72	N.A.	−0.65
Ponferrada	−0.82	−0.34	−0.97	−1.39
Salamanca	−0.06	−0.26	−0.09	−1.53
Segovia	−0.83	0.22	−0.37	−0.95
Soria	−0.17	−0.18	0.30	−0.80
Valladolid	N.A.	−0.28	−0.09	−1.70
Zamora	N.A.	−0.10	−0.75	N.A.
Total	−0.15	−0.05	−0.05	−0.88

**Table 2:** Trends for maximum and minimum temperature, precipitation, and snow days for the stations studied, between 1960 and 2011.

Station	*T* max			*T* min			Precipitation			Snow days		
	Test *Z*	Signific.	*Q*	Test *Z*	Signific.	*Q*	Test *Z*	Signific.	*Q*	Test *Z*	Signific.	*Q*
Burgos (Villafría)	2.13	∗	0.029	1.24		0.037	−0.91		−0.98	−0.76		−0.064
Valladolid	2.47	∗	0.029	2.42	∗	0.056	−1.46		−1.546	−1.58		−0.05
Zamora	3.56	∗∗∗	0.047	0.31		0.006	−0.39		−0.476	−1.23		−0.025
Soria	2.33	∗	0.024	−0.24		−0.006	−0.53		−0.614	−1.23		−0.069
León	0.45		0.005	0.35		0.005	−1.6		−2.025	−3.01	∗∗	−0.142
Ponferrada	3.14	∗∗	0.035	−1.13		−0.016	−0.08		−0.16	−2.47	∗	−0.044
Salamanca	3.21	∗∗	0.044	−0.73		−0.013	−1.22		−0.954	−2.32	∗	−0.079
Palencia	0.32		0	2.55	∗	0.008	0.41		0	NaN		
Segovia	NaN			−0.81		−0.018	0.54		0.477	2.032	∗	0.08
Ávila	NaN			NaN			2.75	∗∗	2.247	NaN		

Mann-Kendall test (*Z*) indicates sign of observed trend, with significance of 0.001 (∗∗∗), 0.01 (∗∗) 0.05 (∗). *Q* represents trend slope, using Sen's slope estimator.
